# FOUND Trial: randomised controlled trial study protocol for case finding of obstructive sleep apnoea in primary care using a novel device

**DOI:** 10.1136/bmjopen-2024-090000

**Published:** 2024-07-25

**Authors:** Michelle A Miller, Ly-Mee Yu, Asad Ali, Patricia Apenteng, Peter Auguste, Jeremy Dale, Kath Hope, Milensu Shanyinde, Jenna Grabey, Emma Scott, Anne Smith, Francesco P Cappuccio, the FOUND Trial study group, J Guest, A Hare, I Mcleod, T J Peters, C Rogers, P S Sever, N Siriwardena, J Harris, T M MacDonald, T Quinnell, C Taggart, P Auguste, P Apenteng, F P Cappuccio, C Carr, J Dale, O Dessi, K Hope, O Llion, M Miller, S Prakash, J Rahman, E Scott, A Smith, L-M Yu, E Buckingham, J Chalk, J Grabey, A Grove, R Harrison, M Shanyinde, L Costello, A Ali, S Elwell

**Affiliations:** 1 Warwick Medical School, University of Warwick, Coventry, UK; 2 Nuffield Department of Primary Care Health Sciences, University of Oxford, Oxford, UK; 3 Respiratory and Sleep Sciences, University Hospitals Coventry and Warwickshire NHS Trust, Coventry, UK; 4 Primary Care Clinical Sciences, University of Birmingham, Birmingham, UK; 5 Hope2Sleep, East Yorkshire, UK; 6 University Hospitals Coventry and Warwickshire (UHCW), Coventry, UK

**Keywords:** SLEEP MEDICINE, Randomized Controlled Trial, Primary Health Care, RESPIRATORY MEDICINE (see Thoracic Medicine), HEALTH ECONOMICS

## Abstract

**Introduction:**

Obstructive sleep apnoea (OSA) is a common, but underdiagnosed, sleep disorder. If untreated, it leads to poor health outcomes, including Alzheimer’s disease, cancer, cardiovascular disease and all-cause mortality. Our aim is to determine the feasibility and cost-effectiveness of moving the testing for OSA into general practice and how general practitioner (GP)-based screening affects overall detection rates.

**Methods and analysis:**

Randomised controlled trial of case finding of OSA in general practice using a novel Medicines and Healthcare products Regulatory Agency-registered device (AcuPebble SA100) compared with usual care with internal feasibility phase. A diverse sample of general practices (approximately 40) from across the West Midlands Clinical Research Network will identify participants from their records. Eligible participants will be aged 50–70 years with body mass index >30 kg/m^2^ and diabetes (type 1 or 2) and/or hypertension (office blood pressure >145/90 mm Hg or on treatment). They will exclude individuals with known OSA or chronic obstructive pulmonary disease, or those they deem unable to take part. After eligibility screening, consent and baseline assessment, participants will be randomised to either the intervention or control group. Participants in the intervention arm will receive by post the AcuPebble sleep test kit. Those in the control arm will continue with usual care. Follow-up questionnaires will be completed at 6 months. The study is powered (90%) to detect a 5% difference and will require 606 patients in each arm (713 will be recruited to each arm to allow for attrition). Due to the nature of the intervention, participants and GPs will not be blinded to the allocation.

**Outcomes:**

Primary: Detection rate of moderate-to-severe OSA in the intervention group versus control group. Secondary: Time to diagnosis and time to treatment for intervention versus control group for mild, moderate and severe OSA; cost-effectiveness analysis comparing the different testing pathways.

**Ethics and dissemination:**

The trial started on 1 November 2022. Ethical approval was granted from the South Central Oxford A Research Ethics Committee on 9 June 2023 (23/SC/0188) (protocol amendment version 1.3; update with amendment and approval to renumber to V2.0 on 29 August 2023). Patient recruitment began on 7 January 2024; initial planned end date will be on 31 April 2025.

Results will be uploaded to the ISRCTN register within 12 months of the end of the trial date, presented at conferences, submitted to peer-reviewed journals and distributed via our patient and public involvement networks.

The University of Warwick will act as the trial sponsor. The trial will be conducted in accordance with the Sponsor and Primary Care Clinical Trials Unit standard operating procedures.

**Trial registration number:**

ISRCTN 16982033.

**STRENGTHS AND LIMITATIONS OF THIS STUDY**

- This study uses a randomised controlled trial protocol which is written in accordance with the Standard Protocol Items: Recommendations for Interventional Trials.
- The study will examine multiple outcomes, including effectiveness of case finding for obstructive sleep apnoea (OSA) in general practice, time to diagnosis in both arms and associated full health economic analysis.
- The study uses a novel OSA testing device which is automatic and intended to speed up testing and analysis time.
- The study is limited in that only those individuals currently defined as being at high risk of OSA (obese with hypertension and/or diabetes) are eligible to take part.
- Due to the nature of the study, general practitioners and patients cannot be blinded to the randomisation.

## Introduction

### The problem

 Obstructive sleep apnoea (OSA), often referred to as obstructive sleep apnoea/hypopnoea, is a common sleep disorder, which if untreated leads to poor health outcomes, including Alzheimer’s disease, cancer, cardiovascular disease (CVD) and all-cause mortality.^1^,^3^ Although in the UK it is estimated that 1.5 million adults are living with OSA, up to 85% of cases remain undiagnosed.^4^ Characterised by repetitive partial or complete blockages of the airway during sleep, it leads to interruptions in breathing, raised heart rate, raised blood pressure (BP), blood oxygen desaturation and arousals. In about 60% of patients, OSA is associated with excessive daytime sleepiness (EDS) and is known as obstructive sleep apnoea syndrome (OSAS).^5^ OSAS has a negative impact on quality of life and increases the risk of road traffic accidents (RTAs) by 1.3–7 times.^6^

Recent National Institute for Health and Care Excellence (NICE) guidelines recognise the need for improved recognition, diagnostic testing and treatment for this condition, with testing being offered to all people with suspected OSA.^7^ However, there is no systematic approach to identifying patients with OSA in the general population. Not all patients with OSA experience characteristic symptoms, including poor sleep quality, snoring, impaired alertness, cognitive impairment, nocturia, morning headaches, sexual dysfunction and EDS.^8^ Many rely on their partner’s observations of breathing during sleep, and 42% of people who snore or whose partner snores have not heard of OSA and would not discuss these symptoms with their general practitioner (GP).^4^ In the UK, suspected cases are currently diagnosed with sleep studies through specialist hospital referrals but there is a mismatch between healthcare requirements and sleep service delivery.^9^ For example, at the University Hospital Coventry and Warwickshire National Health Service (NHS) Trust, prior to the pandemic, the time to treatment was much longer than 4 months. Nationally, the backlog of patients awaiting a sleep study was exacerbated during the pandemic, with many sleep service clinical leads being redeployed to COVID-19 duties and sleep testing protocols redesigned.^10 11^

This study aims to address the need for high-throughput COVID safe testing by evaluating the feasibility and effectiveness of a novel OSA diagnostic device in a general practice setting with individuals who are at high risk for OSA.

### Why is this research important?

Treatment of moderate-to-severe OSA with continuous positive airway pressure (CPAP) improves the health and well-being of patients and the management and control in those with moderate-to-severe hypertension. It reduces CVD risk markers^12^ and associated adverse CVD outcomes.^13^ CVD accounts for 42% of deaths in people with untreated OSA compared with 26% of people without it.^14^ Healthcare costs associated with CVD are high and could be reduced by increased detection and treatment of moderate-to-severe OSA.^4 15^ EDS accounts for ~20% of all RTAs; many of these involve drivers with undiagnosed OSAS and hence might be preventable. In a national survey, at the time of OSA diagnosis, 22% had been doing a job requiring them to drive regularly (27% professionally) and 11% had fallen asleep driving.^4^ As well as personal costs, each fatal accident costs society around £1.5 million.^4^

### Review of the evidence

In OSA, complete closure (obstruction) of the airway during sleep stops airflow (apnoea), whereas partial obstruction decreases airflow (hypopnoea) resulting in episodes of brief awakening from sleep (arousals) to restore normal breathing.^16^ The number of apnoeas/hypopnoeas per hour of sleep defines the apnoea-hypopnoea index (AHI) and gives an indication of disease severity.^17^ In 1993, the prevalence of OSA was 4% in middle-aged men and 2% in middle-aged women (ages 30–60 years).^14^ In 2019, Benjafield *et al* estimated that 425 million adults aged 30–69 have moderate-to-severe sleep apnoea globally.^18^ In a study published in 2021, using the Clinical Practice Research Datalink, which represents 8.5% of primary care practices in the UK, the prevalence of OSA in obese patients was 8.4% in men and 3.7% in women.^19^ Male sex, high body mass index (BMI), hypertension and diabetes were the most common risk factors.^19^ The new NICE guidance recognises that OSA is also highly prevalent in individuals with other conditions like polycystic ovary syndrome, atrial fibrillation and hypothyroidism.^7^ Higher prevalence rates have been reported in some high-income countries.^20 21^ OSA is associated with both type 1 and type 2 diabetes^19 22 23^ and increased BMI,^19 24^ and is a frequent cause of resistant hypertension.^25^,^27^

Current NICE guidelines recommend that adults who are sleepy while driving or working with machinery, are employed in hazardous occupations (eg, pilot, bus or lorry driver) or show signs of respiratory or heart failure (with symptoms suggestive of OSA) should be referred urgently to sleep centres.^28^ However, GPs may not always ask sleep-related questions, and information received may not be followed up or acted on.^29^ Indeed, 20% of people with OSA had visited their GP on three or more occasions with symptoms and no action was taken in 11%.^4^

The most common form of treatment is CPAP,^2^ although other treatments are used.^30^ Treating moderate-to-severe OSA can generate health benefits and improve the quality of life of patients, especially those with OSAS who experience an improvement in EDS, and a reduction in risk of RTAs.^31 32^ CPAP is also an effective management in patients with resistant hypertension.^4^

In 2008, NICE appraised the use of CPAP compared with lifestyle management and dental devices for the treatment of adults with moderate-to-severe OSA. It concluded that CPAP is cost-effective with a cost per quality-adjusted life-year (QALY) gained below £5000.^3^ Following a more recent evidence review, NICE guidelines (2021) currently suggest that people with mild OSAS (ie, those with EDS) should also be offered treatment using a fixed-level CPAP.^7^ It also recommends that people’s sleep history should be assessed in individuals with two or more of nine listed common features of OSA which include snoring, witnessed apnoeas and unrefreshing sleep.^7^

Few studies have attempted a targeted case finding approach for OSA in primary care. In Canada, researchers asked family doctors to identify patients at high risk (BMI≥30, type 2 diabetes, treated hypertension or ischaemic heart disease). The prevalence of undetected OSA was high (71% had mild OSA defined as AHI ≥5 to <15/hour; 33% had moderate AHI defined as ≥15 to <30/hour; 16% had severe AHI defined as ≥30/hour).^33^ However, a suggestion that OSA screening could move into general practice has not been formally tested or evaluated in the UK.^34 35^

Current practice varies across the UK with complex referral pathways; some sleep services offer a simple overnight oxygen test (oximetry) as a first-line test; others offer home respiratory polygraphy (RP). Polysomnography is rarely used, requiring overnight admission to a secondary care sleep facility. For example, in Coventry and Warwickshire, patients referred by GPs are currently required to visit a hospital to be trained how to use the sleep study device (eg, NOX T3 (ResMed)). This takes the form of a 30 min appointment with further written and online instructions for patients to follow. Having slept overnight with the device’s set of wired sensors at home, the patient brings the equipment back to the sleep centre for the data recorded to be uploaded and analysed. The multiple arrays of equipment must then be decontaminated with respect to COVID-19 procedures. Around 15–20% of sleep tests need repeating due to incorrect sensor placement.^36^ A sleep/respiratory specialist spends about 2 hours manually scoring each test for diagnosis.

Implementation of the latest NICE recommendations in England will substantially raise the number of people being referred to sleep services. There is, therefore, an even greater and more urgent need for a new, rapid and cost-effective way to diagnose moderate-to-severe OSA, to produce health gains from the use of effective treatments and to tackle the increasing waiting lists for diagnosis.

### A new medical device

**Figure 1:**
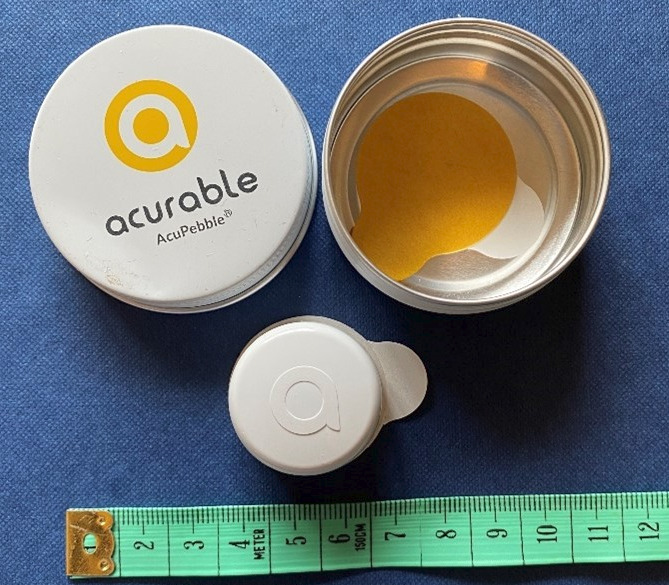
AcuPebble SA100 device (scale in centimetre). Image created by M A Miller.

The Medicines and Healthcare products Regulatory Agency (MHRA)-registered AcuPebble SA100 (referred to as AcuPebble) from Acurable provides a simple and potentially cost-effective option. Its patented technology derives from over 10 years of research at Imperial College London^37^ (see figure 1).

It is clinically validated and has been found to be acceptable to patients.^38^ It is equivalent to current ambulatory gold standard (multichannel RP with manual specialist interpretation) based on recommended American Academy of Sleep Medicine AHI criteria with a positive predictive value of 94.4% and a negative predictive value of 95.83%.^36^ The system is easy to use; 100% of 150 patients recruited for the clinical validation were able to follow the simple accompanying instructions.^38^ The AcuPebble is equivalent to home RP, the NICE-recommended test, with the additional benefits to the patient that it can be deployed in a faster and COVID-secure way. No hospital attendance is required, it is posted to patients and its use does not require training, saving patients’ time and travel costs. The test is comfortable, non-invasive, using a small device attached to the neck over the throat and so allows more natural sleep, without being attached to leads or wires.

There are benefits for healthcare providers and the NHS. Staff no longer need to prepare equipment, train patients or manually interpret recorded signals. The AcuPebble SA100 employs fully automated diagnosis, and its ease of use can release over 1500 hours annually of clinical staff time (based on an average unit seeing 1000 patients annually) (Acurable in-house data). The number of tests that need repeating is significantly lower (less than 1%) than the current approaches to home testing, so helping to cut the excessive waiting times to achieve the recommended 4-week referral-to-treatment NHS target.^39^

## The Trial

This protocol is written in accordance with the Standard Protocol Items: Recommendations for Interventional Trials. Any amendments to the protocol will be reported in the study article.

Please see online supplemental appendix 1 for a full list of abbreviations used in this protocol.

### Aims and objectives

The aim of this randomised controlled trial is to test the feasibility of moving the testing for OSA into a general practice setting using the AcuPebble device and to trial a targeted moderate-to-severe OSA case finding programme. Our objectives are (1) to determine if using this device would increase the detection of moderate-to-severe OSA in high-risk groups within general practice, (2) to assess the cost-effectiveness of screening for moderate-to-severe OSA with AcuPebble in primary care versus usual care in people at high risk and (3) to compare a new general practice-based route for the diagnosis of moderate-to-severe OSA with the standard hospital-based referral pathway.

## Methods and analysis

### Trial design

We are conducting a multicentre, pragmatic, individually randomised, parallel-group, superiority trial and economic evaluation to determine the effectiveness and cost-effectiveness of using a novel MHRA-registered device (AcuPebble SA100) to detect moderate-to-severe OSA in a high-risk group compared with usual care.

The study includes a 2-month internal feasibility phase, in which ‘Stop-Go’ criteria will be used to evaluate the implementation of AcuPebble SA100, recruitment and adherence to the intervention.

### Study setting and recruitment

Participants will be recruited from participating UK general practices in the West Midlands Clinical Research Network (CRN) region (Warwickshire, West Midlands, Worcestershire, Herefordshire, Shropshire and Staffordshire). General practices will be recruited to include different practice types according to list size (small <6000 to large >12 000 patients), deprivation index, rural/urban location and practice type (group practice, etc). Participating general practices, supported by the local CRN and research team, will search their records to identify and invite eligible patients meeting the inclusion criteria.

### Eligibility

#### Inclusion criteria

Adults aged between 50 and 70 years with BMI≥30 kg/m^2^ as of GP records in the last 3 years AND documented (a) diabetes (type 1 or 2) OR (b) hypertension (office BP>145/90 mm Hg or on treatment) OR (c) both (hypertension and diabetes).

#### Exclusion criteria

Patients with known OSA, with known moderate-to-severe chronic obstructive pulmonary disease and those deemed unable to take part by their GP (eg, terminally ill, unable to give consent, etc). Patients with known allergy to acrylate.

### Intervention arm

Participants randomised to the intervention arm will receive the overnight sleep testing AcuPebble device from Acurable by post. Simple participant instructions, including how and where the device is placed, are given via a dedicated mobile app (phone supplied with app if required). Should the test fail, a new test code will be sent to the participant enabling a repeat test before the device is returned in prepaid addressed envelope. When the test is complete, the test data are automatically uploaded directly to Acurable’s secure platform, allowing them to provide the diagnosis, as validated in their study, using their algorithm.

The sleep study platform will include the Epworth Sleepiness Scale (ESS) and an optional brief questionnaire to assess acceptability of the intervention. The number of completed/valid tests, number of failed tests and returned devices will be an indication of feasibility. Participants are expected to perform the sleep testing within a week of receipt of the AcuPebble device. Participants will be followed up remotely 6 months from the date of randomisation. Data will be collected both from participants and medical notes review.

The results of the sleep studies will be reviewed by our sleep consultant to confirm any diagnoses of OSA. He will notify the participant’s GP of the results and advise whether the patient needs to be referred to the sleep clinic for treatment or further investigation.

### Control arm

Participants randomised to the control arm will continue with usual care provided by their GP. Patients presenting to the GP with symptoms of OSA will be referred for assessment through their local usual care pathway as per the NICE guidelines. Participants will be followed up remotely 6 months from the date of randomisation. Outcome data will be collected both from participants and medical notes review.

### Trial procedures

#### Informed consent

Written informed consent (see online supplemental appendix 2) will be obtained by appropriately trained members of the research team. Potential participants are contacted via post; if they express interest, the research team will explain the study and answer any questions; and if they are willing to continue, they are asked to complete a consent form. The original signed copy will be kept in a locked filing cabinet in the dedicated locked trial office.

##### Baseline assessments

Following informed consent and *before* randomisation, participants will be asked to complete the following questionnaires: EuroQol Heath Status Questionnaire (EQ-5D-5L) with visual analogue scale (VAS)^40^ and Client Service Receipt Inventory (CSRI).

### Randomisation and blinding

**Figure 2:**
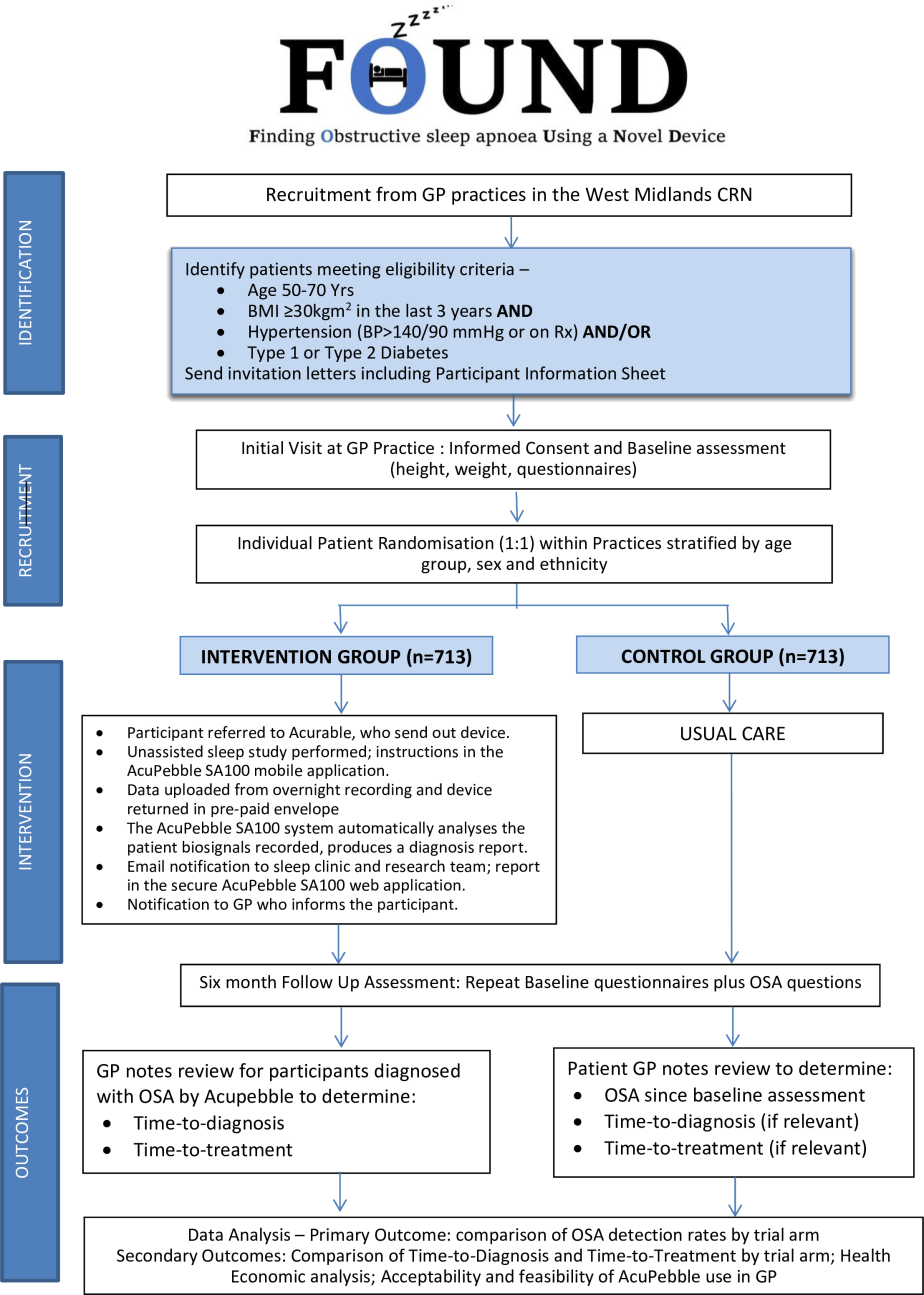
Trial flow diagram. BMI, body mass index; BP, blood pressure; CRN, Clinical Research Network; GP, general practitioner; OSA, obstructive sleep apnoea.

Figure 2 shows the design of the study. Participants will be randomised (1:1) to receive the intervention or usual care using a validated web-based randomisation programme (sortition). Randomisation will be minimised with a non-deterministic minimisation algorithm to ensure site, age (<60/≥60 years), sex (F/M) and ethnicity (White/Other) are balanced across the two groups. Individual randomisation is appropriate because the risk of contamination is very low, since the device is sent to participants directly and the assessment of the primary outcome (diagnosis of moderate-to-severe OSA) will be automated (unbiased).

Due to the nature of the intervention, participants and their GPs will not be blinded to the allocation of intervention. But the primary outcome can be considered blinded as it is fully automated. The statisticians will remain blinded to the allocation when performing data analysis.

### Follow-up

Participants will be followed up at 6 months from point of randomisation. All participants will be asked to complete repeat measures of the EQ-5D-5L with VAS, CSRI and a short OSA questionnaire. Two reminders will be sent, but any participants who have not returned their completed questionnaires within 1 month of the date of posting will be considered non-responders and their questionnaires considered missing.

The GP notes of all participants in the usual care group will be reviewed to identify any diagnoses of OSA since randomisation (primary outcome) and for those with a positive diagnosis, time to diagnosis, time to commencement of treatment and type of treatment prescribed.

For those in the intervention group *diagnosed with OSA* since randomisation, their GP notes will be reviewed to determine time to commencement of treatment and type of treatment prescribed. The GP notes of those who were referred for further investigation will also be reviewed to confirm whether a subsequent diagnosis of moderate-to-severe OSA was made, and if so, details of any treatment started.

Reviewing the GP notes of those in the intervention group who tested negative for OSA and needed no further follow-up is considered unnecessary, as no further information is required for this trial, and hence there is no justification for accessing participants’ medical information. A variety of means of communication will be used to both raise awareness in the practice and to gain a response or to send invitations. Texts and emails will also be used where appropriate.

Any subsequent post-trial visits and treatment by the GP or sleep clinic are not part of the research and represent a return to the usual care pathway after diagnosis.

### Outcomes

#### Primary outcomes

- Participants diagnosed with moderate-to-severe OSA, defined as an AHI reading of 15–30 (moderate) or >30 (severe) episodes per hour.
- The AcuPebble report for those in the intervention group and the GP notes review for those in the usual care group will be reviewed 6 months after randomisation to identify any diagnoses of moderate-to-severe OSA since randomisation.

#### Secondary outcomes

- Time to completion of testing from randomisation, time to diagnosis from randomisation and time to treatment from randomisation for new pathway (intervention group) versus usual care. For those diagnosed with moderate-to-severe OSA (as detailed above for both the intervention and usual care groups) and OSAS, their AcuPebble or GP notes will be reviewed 6 months after randomisation to determine time to completion of testing, time to diagnosis, time to commencement of treatment and type of treatment prescribed. For the usual care pathway, we will also look at the individual components of the usual referral pathway, that is, time to test from referral, time to diagnosis from referral, time to treatment from referral. Date of referral is defined as date when the patient was referred.
- Detection of participants diagnosed with any OSA (mild, moderate, severe), defined as an AHI reading of ≥5 to <15 (mild), ≥15 to <30 (moderate) or ≥30 (severe) episodes per hour for new (intervention) versus usual care pathway. This will be determined from the AcuPebble report for those in the intervention group and the GP notes review for those in the usual care group.
- Detection of participants diagnosed with OSAS, defined as those with OSA (mild, moderate, severe (as above)) and with evidence of EDS (ESS score >11) for new (intervention) versus usual care pathway. This will be determined from the AcuPebble report for those in the intervention group and the GP notes review for those in the usual care group.
- Health-related quality of life associated with new and current pathways. QALYs will be calculated based on information collected from participants at baseline and 6 months using the EQ-5D-5L.
- Cost-effectiveness analysis comparing new and current pathways. All participants will be sent follow-up questionnaires either by email or post (as detailed above); these will include the EQ-5D-5L with VAS, CSRI and information about procedures undertaken to diagnose or treat moderate-to-severe OSA since randomisation. Further data on health service usage will be obtained from the participant notes review undertaken at 6-month follow-up. This review will collect data on service usage related to sleep issues in the 6 months prior to date of randomisation to provide a baseline value and during the 6 months after randomisation to provide follow-up data.
- Feasibility and acceptability of new sleep study testing to be tested against the following criteria in the feasibility phase: (1) the number of GP practices recruited and set up (four required for feasibility; No-Go criteria: only one practice recruited); (2) to recruit 80 patients within 2 months of the first patient recruited (No-Go: <50% recruited); (3) AcuPebble testing successfully implemented; (4) at least 90% intervention completion (No-Go: <50% completion). Participants using the AcuPebble also have the option to complete a brief survey about their experience of using the AcuPebble on the AcuPebble app, following the completion of their sleep study. These are standard questions currently asked and analysed by Acurable for all AcuPebble users.

### Schedule of delivery of intervention and data collection

The trial events’ schedule and data assessments are summarised in table 1.

**Table 1:** Trial schedule of events and data collection

	Before randomisation	Baseline+randomisation	Intervention group	6 months
	Remote	Face to face	Remote by Acurable	Remote
Identification of eligible patients (inclusion/exclusion criteria)	✓			
Invitation to participate in study	✓			
Written and witnessed informed consent		✓		
Collection of questionnaires (Clinical and Health Economics)		✓		✓
Randomisation		✓		
Delivery of AcuPebble			✓	
Analysis of OSA test and reporting outcome			✓	
Primary outcome				✓
Secondary outcomes				✓

OSAobstructive sleep apnoea

### Discontinuation/withdrawal of participants from study

If the participant wishes to withdraw from follow-up, we will use their data up to the point that they discontinue from the trial. No participants will be replaced if they are discontinued or withdraw. Participants will only be withdrawn from the intervention by the research team should the intervention be deemed unsafe, or withdrawn from the trial if participant subsequently found to be ineligible. Reasons for withdrawal will be captured and recorded in the trial database.

### End of study

The end of the trial is the final data capture of the last participant’s GP notes review.

### Adverse event management

The safety reporting window for the trial will be defined as the period between randomisation and 6-month follow-up of each participant in the trial.

This trial will only collect adverse events (AEs) potentially related to the AcuPebble device. Hence, participants will be encouraged to self-report any AEs and serious adverse events in the 4 weeks following randomisation directly to the trial office and will be reviewed and reported on as per the Good Clinical Practice (GCP).

### Data management

Details of the data management procedure are documented in a trial-specific data management plan reviewed and signed by all applicable parties prior to the first participant being enrolled. The data management will be run in accordance with the Primary Care Clinical Trials Unit (PC-CTU) standard operating procedures (SOPs), which are fully compliant with GCP. The trial will comply with the UK General Data Protection Regulation (GDPR) and Data Protection Act 2018, which require data to be anonymised as soon as it is practical to do so.

AcuPebble SA100 is a registered NHS device and data will be stored in the UK. The AcuPebble data will be downloaded in .csv format from the AcuPebble SA100 web application, and then uploaded to the clinical database that is managed and hosted by the University of Oxford PC-CTU. Pseudonymised study data, only accessible by relevant members of the data management team, are stored on regularly backed-up, VPN secure network drives in accordance with the GDPR and participants’ consent.

### Data sharing plan

Due to the sensitive nature of the medical data collected for this trial (ie, individual risk factors and disease diagnosis), the full data set will not be placed in a public access repository but will be available, on request, from the lead author.

### Sample size determination

The detection rate of new cases of moderate-to-severe OSA using usual care is estimated at <5% per year. Given the previously observed rates of hypertension (39%), obesity (34%) and diabetes mellitus (15%) in individuals with OSA, we would expect that the targeted case finding intervention would yield a detection rate of 10% or more in the high-risk groups. The study has been powered (90%) at 5% two-sided significance level to detect a 5% difference in rate of new cases of moderate-to-severe OSA between intervention and usual care. The study will require 606 participants in each arm. To allow for 15% attrition and lost to follow-up, it is anticipated that 713 in each arm need to be recruited.

## Statistical methods

Feasibility outcomes will be assessed descriptively and reported to the data monitoring and safety committee and trial steering committee.

The study results will be reported in accordance with the Consolidated Standards of Reporting Trials 2010 reporting guidelines (www.consort-statement.org/downloads/consort-statements) and a statistical analysis plan will be prepared before recruitment starts.

Baseline variables will be presented by randomised group descriptively. Trial results presented as comparative summary statistics with 95% CIs. All tests will be done at a 5% two-sided significance level.

The primary analysis will include all randomised participants, as defined by protocol eligibility criteria, regardless of what intervention they actually received or compliance of intervention. The primary analysis of the primary outcome (detection of moderate-to-severe OSA) will be performed using a logistic generalised linear mixed effects model, adjusting for minimisation factors (age, sex and ethnicity) as fixed effect and general practices as random effect. A similar approach will be used for other secondary binary outcomes. Time-to-event outcomes will be analysed using a Cox regression model with similar adjustment as the primary analysis. Secondary analysis of time to starting treatment will include the primary analysis population who were diagnosed with moderate-to-severe OSA and individuals with evidence of EDS/OSAS. Participants not starting treatment will be censored at last contact date.

Missing data will be reported and the missing data pattern will be explored. Additional sensitivity analysis using imputation methods will be performed. Safety outcomes of the intervention will be descriptive.

## Health economic evaluation

A trial-based economic analysis will be undertaken to assess the cost-effectiveness of the use of AcuPebble in general practice compared with GP referral to hospital for the diagnosis of people with moderate-to-severe OSA. Information about resource use will be collected using resource use questionnaires and notes review, which will include equipment (AcuPebble and home sleep RP) required, staff required and healthcare resource while participants are awaiting referral and treatment. Resource use will be valued using national sources.^41^

Additionally, we will conduct a systematic literature review to identify existing economic evidence regarding OSA diagnosis and in individuals with evidence of EDS/OSAS and treatment. Insights from this and input from clinical experts will inform the model structure. In the model, we will include a ‘no screening’ strategy to model the natural history of people living with OSA, in addition to the two screening approaches: using AcuPebble in general practice and GP referral to hospital-based standard care (home sleep RP). We envisage the economic model will comprise two stages. In the first stage, we will use a decision tree illustrative structure to model the short-term costs and benefits associated with identifying people living with OSA and in individuals with evidence of EDS/OSAS following screening. In the second stage, we will use a state transition Markov structure to model the progression of events associated with moderate-to-severe OSA (eg, stroke, CVD, myocardial infarction), and RTAs in those with individuals with evidence of EDS/OSAS, then the long-term costs and benefits associated with treatment following the screening/no screening strategies.

The economic model will require clinical and cost inputs related to the strategies. Clinical inputs (eg, time to test, time to diagnosis and time to treat) will be obtained from the clinical trial and supplemented with information from the literature (eg, rate of moderate-to-severe people with OSA experiencing a stroke, associated costs and utility values). The cost associated with using AcuPebble will be obtained from Acurable and costs for GP referral to hospital-based standard care will encompass all resource use and costs for GP referral using the home sleep RP. Resource use questionnaire will be developed using the CSRI to capture healthcare associated with diagnosis and patient management while awaiting referral and treatment (eg, healthcare professional visits, inpatient/outpatient visits and medication) with information collected at baseline and 6 months. Resource use will be valued using unit costs from national sources. Where costs are not available, these will be obtained from published literature and adjusted using appropriate indexes. All costs included will be those directly related to the UK NHS and personal social services.

Outcomes in the form of QALYs will be calculated using data collected from the EQ-5D-5L. The health-related quality of life information collected will enable us to estimate the short-term impact of being screened on the participants’ quality of life. The EQ-5D is a widely used generic measure of health-related quality of life that enables the calculation of QALYs and is recommended for use in economic evaluations in healthcare.^42^ The EQ-5D-5L will comprise the descriptive section and the accompanying VAS, which ask participants about their quality of life on a specific day.

In line with NICE recommendations,^42^ costs incurred and benefits accrued will be discounted at 3.5% per annum, and the findings will be presented as an incremental cost-effectiveness ratio in terms of costs per additional life-year and QALY associated with each of the screening options over a lifetime horizon. Additionally, we will present results to show the short-term (eg, additional cases of moderate-to-severe OSA and in individuals with evidence of EDS/OSAS detected following each screening strategy) and the long-term benefits of screening and treatment (eg, reduction in strokes, CVDs and myocardial infarction). Several sensitivity and scenario analyses will be undertaken to estimate the impact to the base case cost-effectiveness results. The model will form the basis for conducting value of information analysis, which will quantify the total expected cost due to the remaining uncertainty around the cost-effectiveness of introducing screening for OSA/OSAS.

## Oversight, monitoring and quality assurance

The trial will be conducted in accordance with the current approved protocol, GCP, relevant regulations, the Sponsor and Oxford PC-CTU’s SOPs and study-specific working instructions. All principal investigators, coordinating centre staff and site staff will receive training in trial procedures according to GCP where required. Regular monitoring will be performed according to GCP using a risk-based approach. Data will be evaluated for compliance with the protocol and accuracy in relation to source documents where possible.

The composition, roles and responsibilities of various management committees are detailed in their respective charters. These include the *Trial Management Group* (*TMG*) which will be responsible for the monitoring of all aspects of the trial’s conduct and progress and will ensure that the protocol is adhered to, and that appropriate action is taken to safeguard participants and the quality of the trial itself, and the *Research Steering Group* (*RSG*) which will provide oversight of the research and will operate as the key forum through which the funder shall be informed as regards progress and outcomes. An independent *Trial Steering Committee* (*TSC*) will provide an overall supervision of the trial and ensure it is being conducted in accordance with the principles of GCP. An independent *Data Monitoring and Ethics Committee *will review and monitor the accruing data to ensure the rights, safety and well-being of the trial participants. An *Innovation and Implementation Monitoring Team* will be established to assist Acurable in the development and commercialisation of the product to this new market—GPs/primary care.

## Patient and public involvement

The study has collaboratively involved patients in the design and delivery of the research. The research proposal was developed with input from the founder of Hope2Sleep and the managing secretary of the Sleep Apnoea Trust Association. Once the study was funded, a third patient was recruited to form a patient and public involvement (PPI) panel. The role of the PPI panel is to work with the PPI lead to ensure that the patient perspective is taken into consideration throughout the study. For example, the PPI group contributed to the development of the study protocol and study documentation and are actively involved in the study management committees (TMG, TSC, Research Steering Committee). They will be instrumental in the interpretation of study findings and ensuring that the findings reach a wide range of people.

All involvement activities will align with the UK Standards for Public Involvement, and training and support will be provided where necessary. The public contributors will be offered honoraria and expenses in line with recommendations from the National Institute for Health and Care Research (NIHR) Centre for Engagement and Dissemination. The PPI lead will be responsible for capturing the impact of involvement throughout the project and reporting activities using the Guidance for Reporting Involvement of Patients and the Public framework.^43^

## Ethics and dissemination

### Sponsor and governance arrangements

The University of Warwick will act as trial sponsor (SOC.09/22-23; sponsorship@warwick.ac.uk), which will be conducted by the Oxford PC-CTU. The trial will be conducted in accordance with the Sponsor and PC-CTU’s SOPs. The study sponsor and funders have no influence or authority over the study design, data collection, analysis, reporting, etc.

###  Ethical approvals and reporting

The investigators will ensure that this trial is conducted in accordance with the principles of the Declaration of Helsinki and with GCP.

Ethical approval was granted from the South Central Oxford A Research Ethics Committee on 9 June 2023 (23/SC/0188) (protocol amendment version 1.3; update with amendment and approval to renumber to V2.0 on 29 August 2023).

The chief investigator will submit and, where necessary, obtain approval from the above parties for all substantial amendments to the original approved documents. All approved protocol amendments will be conveyed to the Trial investigators and, where appropriate, participants.

The CI (or delegate) shall submit throughout the clinical trial, or on request, progress report to the Research Ethics Commitee (REC) (where required), the Health Research Authority (where required), the funder (where required) and the Sponsor (where required). In addition, an end of trial notification and final report will be submitted to the REC and the Sponsor.

## Trial registration

Prior to the recruitment of the first participant, the trial was registered on the ISRCTN Database (16982033). Results will be uploaded to this register within 12 months of the end of the trial date.

### Trial start and end dates

The trial started on 1 November 2022. Patient recruitment began on 7 January 2024. Initial planned end date will be on 31 April 2025.

### Notification of serious breaches to GCP and/or trial protocol

The management of non-compliances will be informed by the Oxford PC-CTU’s SOPs.

### Trial protocol deviation and violations

A trial-related deviation is a departure from the ethically approved trial protocol or other trial document or process (eg, consent process or administration of trial intervention) or from GCP or any applicable regulatory requirements. Any deviations from the protocol will be documented in a protocol deviation form and filed in the trial master file.

A PC-CTU’s SOP is in place describing the procedure for identifying non-compliances, escalation to the central team and assessment of whether a non-compliance/deviation may be a potential serious breach.

### Indemnity

The University of Warwick has a specialist insurance policy in place which would operate in the event of any participant suffering from harm because of their involvement in the study on Zurich Municipal Insurance. NHS indemnity operates in respect of the clinical treatment that is provided.

### Risk assessment and study monitoring

A risk assessment and monitoring plan was prepared before the study opened and will be reviewed as necessary over the course of the trial to reflect significant changes to the protocol or outcomes of monitoring activities. Monitoring will be coordinated by the PC-CTU quality assurance manager or delegate. The level of monitoring required will be informed by the risk assessment.

### Dissemination plans

Trial results will be first reported to the trial collaborators. The statistical report will be prepared by the trial statistics team and will be incorporated into the final trial report which will be drafted by the trial team. The final version will be reviewed and agreed by the TSC and RSG before submission to the NIHR. The main findings of the study as well as specific articles with regard to the health economic assessment, for example, will be written up and submitted to a journal for publication. Findings will be submitted for presentation at relevant scientific conferences. Updates and recruitment numbers are updated on our websites (see FOUND — Oxford University - Primary Care Clinical Trials Unit and FOUND Trial (warwick.ac.uk))). Regular research updates are submitted to Researchfish (Ttrack research and evidence impact with Researchfish by Interfolio).

Awareness of OSA will be increased within the primary care community and public by dissemination of study findings through the Hope2Sleep network (25 000 members), Sleep Apnoea Trust (~5000 members), scientific meetings and media engagement. A stakeholder engagement dissemination event will be held at the end of the study.

## supplementary material

10.1136/bmjopen-2024-090000 online supplemental file 1

10.1136/bmjopen-2024-090000 online supplemental file 2
