# Supplementary material for: FOUND Trial: randomised controlled trial study protocol for case finding of obstructive sleep apnoea in primary care using a novel device
Source: BMJ Open. 2024 Jul 25;14(7):e090000. doi: 10.1136/bmjopen-2024-090000 (PMC11444078; doi:10.1136/bmjopen-2024-090000)
Supplement: online supplemental file 1 [file bmjopen-14-7-s001.pdf]

Appendix 1: Abbreviations

List of abbreviations/GLOSSARY

Abbreviation	Explanation
AASM	American Academy of Sleep Medicine
AE	Adverse event
AHI	Apnoea–Hypopnoea Index
BMI	Body Mass Index
BP	Blood Pressure
CI	Chief Investigator
CONSORT	Consolidated Standards of Reporting Trials
COPD	Chronic Obstructive Pulmonary Disease
CPAP	Continuous Positive Airway Pressure
CPRD	Clinical Practice Research Datalink
CSRI	Client Service Receipt Inventory
CVD	Cardiovascular Disease
DMP	Data Management Plan
EDS	Excessive Daytime Sleepiness
ESS	Epworth Sleepiness Scale
EQ-5D-5L	EuroQol Heath Status Questionnaire
GCP	Good Clinical Practice
GP	General Practitioner
ISRCTN	International Standard Randomised Controlled Trial Number
MHRA	Medicine and Healthcare products Regulatory Agency
NHS	National Health Service
NICE	National Institute of Health and Care Excellence
NIHR	National Institute for Health and Care Research
NPV	Negative Predictive Value
OSA	Obstructive Sleep Apnoea
OSAH	Obstructive Sleep Apnoea/Hypopnoea

OSAS	Obstructive Sleep Apnoea Syndrome (OSA + EDS)
PC-CTU	Oxford Primary Care Clinical Trials Unit
PI	Principal Investigator
PPI	Patient & Public Involvement
PPV	Positive Predictive Value
PSG	Polysomnography
QALY	Quality-adjusted life year
RP	Respiratory Polygraphy
RTA	Road Traffic Accident
SAE	Serious Adverse Event
SOP	Standard Operating Procedure
TSC	Trial Steering Committee
UHCW	University Hospitals Coventry & Warwickshire
VAS	Visual Analogue Scale
