# Supplementary material for: FOUND Trial: randomised controlled trial study protocol for case finding of obstructive sleep apnoea in primary care using a novel device
Source: BMJ Open. 2024 Jul 25;14(7):e090000. doi: 10.1136/bmjopen-2024-090000 (PMC11444078; doi:10.1136/bmjopen-2024-090000)
Supplement: online supplemental file 2 [file bmjopen-14-7-s002.pdf]

FOUND

Finding Obstructive sleep apnoea Using a Novel Device

Trial title: Case finding of obstructive sleep apnoea in primary care using novel device: a randomised controlled trial - FOUND

INFORMED CONSENT FORM

REC Number: 23/SC/0188 **IRAS Number: 323422**

Chief Investigators: Dr Michelle A. Miller; Professor Francesco P. Cappuccio

Participant ID:

If you agree, please initial box:

1. I confirm that I have read the Participant Information Sheet dated ___ ___ ___ (version___) for this trial. I have had the opportunity to consider the information, ask questions and have had these answered satisfactorily.	
2. I understand that my participation is voluntary and that I am free to withdraw at any time without giving any reason, without my medical care or legal rights being affected.	
3. I understand that data collected during the trial may be looked at by the FOUND study team for research purposes.	
4. I give permission for authorized members of the FOUND study team to have access to the relevant sections of my GP medical records.	
5. I understand that I will be randomised to receive either usual care called the control group or use of the testing AcuPebble device, called the intervention group and I will not be able to choose which I will receive.	
6. If I am randomized to the intervention group, I consent to my personal contact details being passed to Acurable who are providing the AcuPebble device for the sleep test.	
7. I understand that if I wish to withdraw from follow-up that data which are collected up to the point of my withdrawal will still be used.	
8. I understand that I will be required to provide information to the research team through paper/online questionnaires.	
9. I consent to being contacted by the research team for the purposes of trial follow up (e.g. by email, text message, phone or post) and I understand that this will require me to provide my contact details to the research team.	
10. I agree to take part in this trial.	

Name of Participant	Date	Signature
Name of Person taking Consent	Date	Signature

**1 copy for participant; 1 (original) to trial office in Warwick; 1 (copy) to be kept in medical notes /site file.*

FUNDED BY NIHR | National Institute for Health and Care Research
