# Supplementary material for: FOUND Trial: randomised controlled trial study protocol for case finding of obstructive sleep apnoea in primary care using a novel device
Source: BMJ Open. 2024 Jul 25;14(7):e090000. doi: 10.1136/bmjopen-2024-090000 (PMC11444078; doi:10.1136/bmjopen-2024-090000)
Supplement: Supplementary file 3 [file bmjopen-2024-090000.reviewer_comments.pdf]

PEER REVIEW HISTORY

BMJ Open publishes all reviews undertaken for accepted manuscripts. Reviewers are asked to complete a checklist review form (**http://bmjopen.bmj.com/site/about/resources/checklist.pdf**) and are provided with free text boxes to elaborate on their assessment. These free text comments are reproduced below.

ARTICLE DETAILS

**TITLE (PROVISIONAL)**	The FOUND Trial: Randomised controlled trial study protocol for case finding of obstructive sleep apnoea in primary care using a novel device.
**AUTHORS**	Miller, Michelle; Yu, Ly-Mee; Ali, Asad; Apenteng, Patricia; Auguste, Peter; Dale, Jeremy; Hope, K; Shanyinde, M; Grabey, Jenna; Scott, Emma; Smith, Anne; Cappuccio, Francesco Paolo

This article was not externally reviewed at BMJ Open. Protocols that have been independently assessed prior to submission to BMJ Open are usually fast-tracked to publication on the grounds that further substantial changes will not be possible. This independent assessment will usually be external, independent review for both a substantial grant award from a non-commercial or government funder and ethics approval.
